# Batch-Fabricated α-Si Assisted Nanogap Tunneling Junctions

**DOI:** 10.3390/nano9050727

**Published:** 2019-05-10

**Authors:** Aishwaryadev Banerjee, Shakir-Ul Haque Khan, Samuel Broadbent, Rugved Likhite, Ryan Looper, Hanseup Kim, Carlos H. Mastrangelo

**Affiliations:** 1 Department of Electrical and Computer Engineering, University of Utah, Salt Lake City, UT 84112, USA; devaash88@gmail.com (A.B.); khanshakirul@gmail.com (S.-U.H.K.); rugved.likhite@utah.edu (R.L.); hanseup@gmail.com (H.K.); 2 Department of Chemistry, University of Utah, Salt Lake City, UT 84112, USA; samuelbroadbentnj@gmail.com (S.B.); r.looper@utah.edu (R.L.)

**Keywords:** nanogap electrodes, gold adhesion, IOT, batch fabrication, bio-sensing, molecular junctions, α-Si, quantum tunneling, protein detection

## Abstract

This paper details the design, fabrication, and characterization of highly uniform batch-fabricated sidewall etched vertical nanogap tunneling junctions for bio-sensing applications. The device consists of two vertically stacked gold electrodes separated by a partially etched sacrificial spacer layer of sputtered α-Si and Atomic Layer Deposited (ALD) SiO_2_. A ~10 nm wide air-gap is formed along the sidewall by a controlled dry etch of the spacer. The thickness of the spacer layer can be tuned by adjusting the number of ALD cycles. The rigorous statistical characterization of the ultra-thin spacer films has also been performed. We fabricated nanogap electrodes under two design layouts with different overlap areas and spacer gaps, from ~4.0 nm to ~9.0 nm. Optical measurements reported an average non-uniformity of 0.46 nm (~8%) and 0.56 nm (~30%) in SiO_2_ and α-Si film thickness respectively. Direct tunneling and Fowler–Nordheim tunneling measurements were done and the barrier potential of the spacer stack was determined to be ~3.5 eV. I–V measurements showed a maximum resistance of 46 × 10^3^ GΩ and the average dielectric breakdown field of the spacer stack was experimentally determined to be ~11 MV/cm.

## 1. Introduction

**Figure 1:**
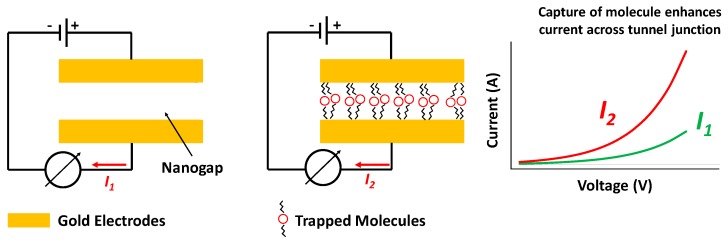
Schematic of tunneling nanogap junctions. Trapping the molecules within the nanogap alters the junction I–V characteristics.

In order to realize robust, uniform, tunable, and Complementary Metal-Oxide-Semiconductor (CMOS)-compatible molecular tunnel junctions which can cater to detection of a variety of organic molecules, sidewall etched nanogap tunneling electrodes were introduced in 2006 [1,2,3]. Essentially, these devices consist of a top and bottom pair of electrodes electrically isolated by a thin insulating dielectric spacer layer. The spacer is partially etched away along the edges, wherein after chemical functionalization, organic molecules end up covalently attached to the electrode pair. These newly attached molecules provide additional electrical pathways for charge conduction between the electrodes. Therefore, this allows for inspection into charge transport across the molecular junction with and without conduction paths introduced by the foreign molecules as illustrated in the schematic of Figure 1, effectively decoupling the electrical characteristics of the covalently bonded molecules and the platform device. The functional molecules (for example a self-assembled-monolayer (SAM) of thiols) can be localized to desired locations between the nanogap electrodes to form the metal-molecule-metal junctions. 

Nanogap junctions have been previously fabricated by mechanical controllable break (MCB) [4], functionalized by organic molecular monolayers sandwiched between a pair of electrodes [5], and tested using STM/AFM tip probing methods [6]. Additionally, nanogap junctions have been previously fabricated by electrochemical methods [7], shadow mask evaporation methods [8], and electromigration [9]. Some of the more recent and exotic methods of nanogap fabrication include template-based nanogap fabrication where molecules and nanostructures are used for engineering the gap [10] and using Focused Ion Beam (FIB) and Oxidative Plasma Ablation for nanogap electrode fabrication [11,12]. While nanogap electrodes fabricated by each of these methods have resulted in extremely valuable results, there are also certain fundamental disadvantages in using them [13]. For example, the MCB method is too cumbersome for high-density circuit applications since it requires macroscopic piezoelectric components for nanogap formation. Electrochemical methods require precise feedback mechanisms in real-time to monitor and accurately fabricate the electrodes and ensure a precise nanogap between them. The oblique angle shadow method requires very low temperature conditions for metal evaporation resulting in small metal grain sizes (thereby ensuring a uniform control of the nanogap between the electrodes). Electromigration essentially requires joule heating to form the nanogap, which means that there is also a high chance of the undesired melting of the metal. In addition, this sometimes leads to the deposition of debris at the critical junction. Other methods involve expensive fabrication techniques like electron beam lithography (EBL) and Molecular Beam Epitaxy (MBE) for consistent results. Furthermore, most of the nanogaps formed using these methods are highly non-uniform in nature, which makes them unsuitable for bio-sensing applications. Sidewall-fabricated nanogap junctions address the majority of the limitations [14] imposed by the above methods; hence they are able to produce nanogap junctions massively with good uniformity characteristics.

In this article, we introduce a new batch-fabrication method of nanogap tunneling junctions and we perform an exhaustive characterization of the uniformity of the spacer layer, inspect tunneling current characteristics, and determine the potential barrier of the thin spacer layer as well as the maximum operating voltage for the device. Molecular devices based on this construction method have been previously used as tunneling chemiresistors for bio sensing [15,16,17,18] and are potential candidates for low-power consumption gas sensing devices [19,20,21,22]. The nanogap electrodes are chemically functionalized by coating them with a self-assemble-monolayer (SAM) of thiol molecules. When the functionalized devices are exposed to the target molecules, they get “captured” by the SAM. The captured molecules form a molecular bridge across the junction producing an augmented electrical transport between the electrodes. Therefore a nanogap structure can be utilized for the electrical detection of bridging target molecules 

Preliminary sensing results have demonstrated that these nanogap electrodes can be effectively used as chemiresistors for label-free electrical detection of both gas and proteins after chemical functionalization [15,17]. The junction resistance, which is typically in the order of giga Ohms, drops by several orders of magnitude upon detection of a chemical target.

## 2. Materials and Methods

### 2.1. Thin-Film Deposition and Characterization

The uniformity and repeatable deposition of the spacer stack is crucial to a high-yield batch-fabrication of the nanogap electrodes. To determine the deposition rate and perform rigorous statistical analysis of the uniformity of these ultra-thin films, we deposited six different thicknesses (3–8 nm) of SiO_2_ on 4-inch Si wafers as described in 2.3 below. Every variant of SiO_2_ thickness was deposited on five wafers each. We then performed thickness measurements on sixty-nine sites on each of these thirty samples. Similarly, different thicknesses of α-Si thin-film was deposited on 4-inch Si wafers. Due to modelling restraints, a layer of 1 μm of thermal SiO_2_ had to be grown on the sample before the α-Si was deposited. Standard optical methods were used to determine the thickness of both the thin films on the n&k Analyzer 1500 D (n&k Technology). AFM measurements were performed on lithographically patterned thin films to experimentally determine their surface roughness as well as that of the substrate.

### 2.2. Device Structure, Design, and Fabrication

**Figure 2:**
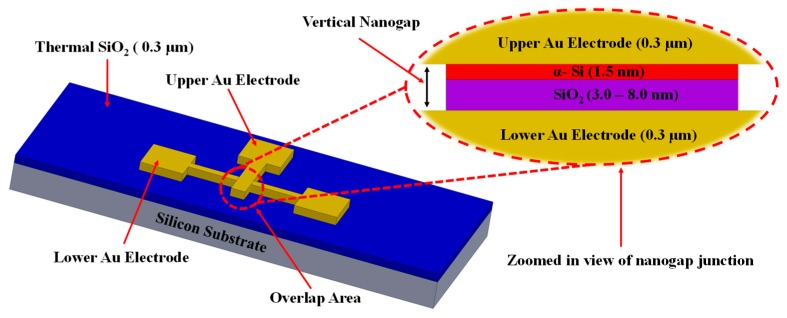
3D schematic of the device of vertical nanogap structure and zoomed-in view of a sacrificial spacer layer.

The nanogap electrode assembly consists of a partially etched spacer film, sandwiched between two thin electrically isolated gold electrodes. The spacer is a sacrificial stack of a very thin dielectric layer of SiO_2_ deposited using a plasma-enhanced ALD method, which provides excellent electrical insulation and an ultra-thin layer of sputtered α-Si, which acts as an adhesive layer between the top gold electrode and the dielectric material. A sacrificial plasma etch of the spacer layer creates a nanogap along the edges of the upper gold electrode. A schematic of the fabricated two-layer nanogap design is shown in Figure 2. Since these nanogap devices will eventually be used for low-power and remote gas-sensing, it is essential that the leakage current during device operation be kept to a minimum so that the parasitic DC power-consumption is extremely low. Since the junction leakage current is directly proportional to the overlap area of the electrodes, an electrode design with a low overlap area should generally ensure a lower leakage current. Therefore, we chose two design architectures having relatively low electrode overlap areas—a “square-overlap” layout (having an overlap area of ~16 μm^2^), which is essentially a perpendicular arrangement of two thin metal wires and a “point-overlap” layout (having an overlap area of ~0.24 μm^2^), which is a very low-overlap arrangement of lithographically patterned pointed tip-ends of patterned electrodes as shown in Figure 8. This also allowed us to investigate into current conduction characteristics as a function of overlap area.

**Figure 3:**
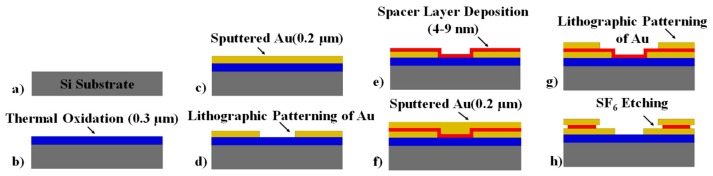
Simplified fabrication process of vertical nanogap electrodes separated by a thin spacer layer. (**a**) 4” Si wafer, (**b**) Thermally grown 300 nm SiO_2_, (**c**) Deposit 200 nm Au bottom electrode and (**d**) pattern lithographically, (**e**) Deposit spacer layer, (**f**) Deposit 200 nm top Au electrode and (**g**) pattern, (**h**) SF_6_ dry etch to form nanogap.

Figure 3 shows the fabrication process of the nanogap electrode assembly. We start by growing ~300 nm of SiO_2_ on a Si wafer (a). This is followed by DC sputtering 25 nm of Cr and 200 nm of Au and subsequent patterning by traditional lithographic techniques to define the lower gold electrodes (b–c). The chemical solution Transene Au etchant TFA was used to selectively etch away the gold. Next, a desired thickness of dielectric material (SiO_2_) was deposited for various time intervals, from 17 to 188 cycles of plasma-enhanced ALD process at a substrate temperature of 200 °C with the commercially available metal-organic precursors tris[dimethylamino]silane (3DMAS) on separate samples to fabricate nanogap electrodes with different spacer thicknesses (e). Then, an ultra-thin layer of α-Si was sputtered for 17 s at 50 W to get a ~1.5 nm film on each sample. Without breaking vacuum, another layer of ~200 nm of Au was sputtered and lithographically patterned to form the top electrodes (f–g). Finally, the samples were dry etched in an inductively coupled plasma etcher (Oxford 100 ICP) with SF_6_ plasma for 40 s at an ICP forward power of 250 W with 45 sccm of SF_6_ flow rate to partially remove the α-Si and SiO_2_, thereby forming a nanogap along the edges of the top electrode (h).

### 2.3. Choice of Spacer Stack: SiO_2_ As the Dielectric Material and Using α-Si As An Adhesive Layer

Since the intended application of the fabricated nanogap sensors is chemical detection and resistance switching at very low standby DC power, the primary requirements of the chosen dielectric material are extremely low leakage current, very high off-resistance, and compatibility with standard CMOS fabrication techniques. Keeping these factors in mind, plasma-enhanced ALD SiO_2_ was chosen as a dielectric material since for a given geometry and thickness of dielectric film, and operating bias voltage, the leakage current is lowest for SiO_2_ films [23].

Since Au is a noble metal, it is chemically inert and does not easily form oxides. Therefore, Au does not adhere well to dielectric films like SiO_2_, which is widely used in CMOS processes. Although the sputtering of Au at elevated temperatures on SiO_2_ substrates is an effective solution [24], the most common practice is to deposit a thin metallic adhesive layer of Cr, Ti, or Ni before depositing the layer of Au. However, such adhesive layers have been known to cause thermal degradation of the film because of grain-boundary diffusion [24], and to ensure a very high off-resistance of the device, using a non-metallic adhesive layer could be a possible solution. Taking this into consideration, we report a novel application of an ultra-thin layer of sputtered α-Si as an effective adhesive layer for Au in microfabrication processes. 

### 2.4. Imaging and Electrical Characterization of Nanogap Electrodes

After fabrication of the devices, high-resolution SEM imaging was done at an accelerating voltage of 15.0 kV by the FEI Nova NanoSEM to inspect the gap formed between the Au electrodes. The I–V characteristics of the nanogap devices were measured on the Keithley 4200A-SCS Semiconductor Parameter Analyzer to ensure that the electrodes were electrically isolated after the nanogap formation and that the resistance between them was substantially high before chemical functionalization and exposure to target analyte. To ensure low-noise and high-fidelity electrical signals, tunneling current measurements were performed in a dark room, inside an electrically shielded enclosure using the Keithley Parameter Analyzer. Instantaneous breakdown voltages of each of these devices were experimentally determined using a simple voltage ramp-up test as described in [22], where the biasing voltage across the Au electrodes was increased at a constant ramp-up rate until the dielectric stack suddenly began to conduct electricity. 

## 3. Results and Discussion

### 3.1. Sacrificial Film Characterization—Thickness Calibration Curves, Uniformity Measurements, and Surface Topology for SiO_2_ and α-Si Thin Films

**Figure 4:**
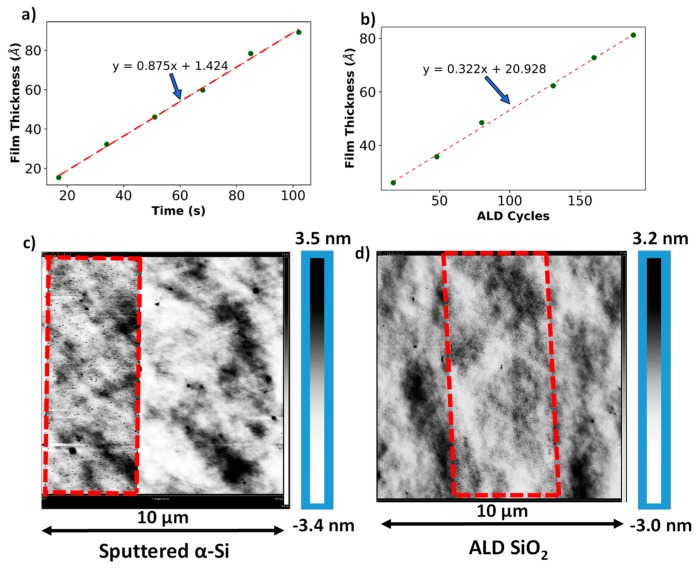
Thickness calibration curves for (**a**) sputtered α-Si and (**b**) ALD SiO_2_, and (**c**) and (**d**) are modified AFM scans showing surface roughness of α-Si ALD SiO_2_ thin films. The red dotted lines outline the features patterned using conventional lithographic techniques.

Figure 4a,b shows an approximately linear deposition rate of ~0.7 Å of SiO_2_ per cycle of plasma-enhanced ALD at 200 °C and a deposition rate of 0.9 Å per second for sputtered α-Si. Figure 4c,d shows AFM images of surface topography of the patterned features of α-Si and ALD SiO_2_ thin films over scan area of 100 µm^2^. The average surface roughness of the α-Si, ALD SiO_2_, and the substrate were measured as ~35 pm, ~34 pm, and ~20 pm. 

**Figure 5:**
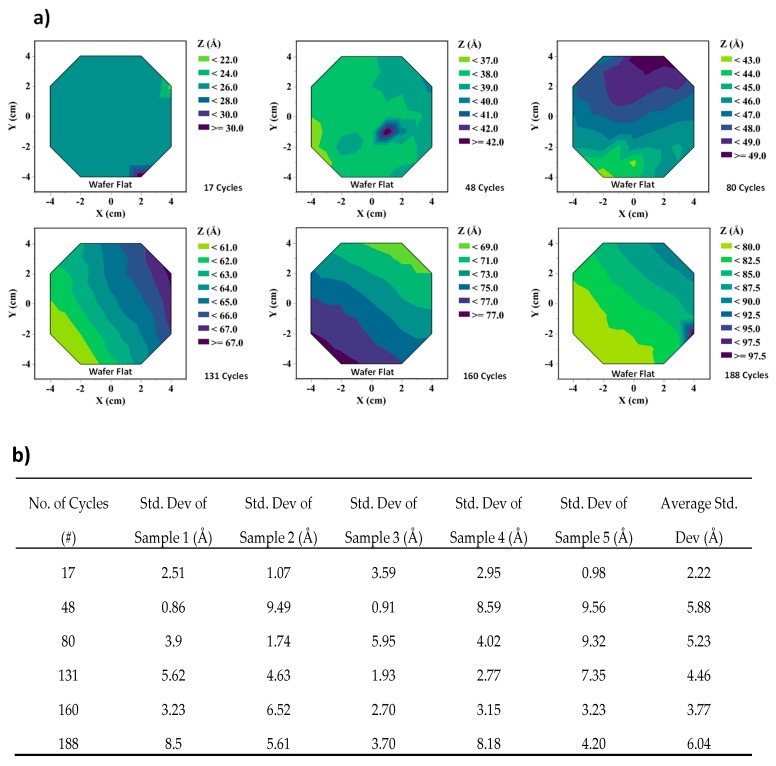
(**a**) Film thickness contour mapping, as measured by optical methods, on 4-inch wafers for different thicknesses of SiO_2_ and (**b**) non-uniformity measurements of different SiO_2_ films done on five samples for each specific thickness.

**Figure 6:**
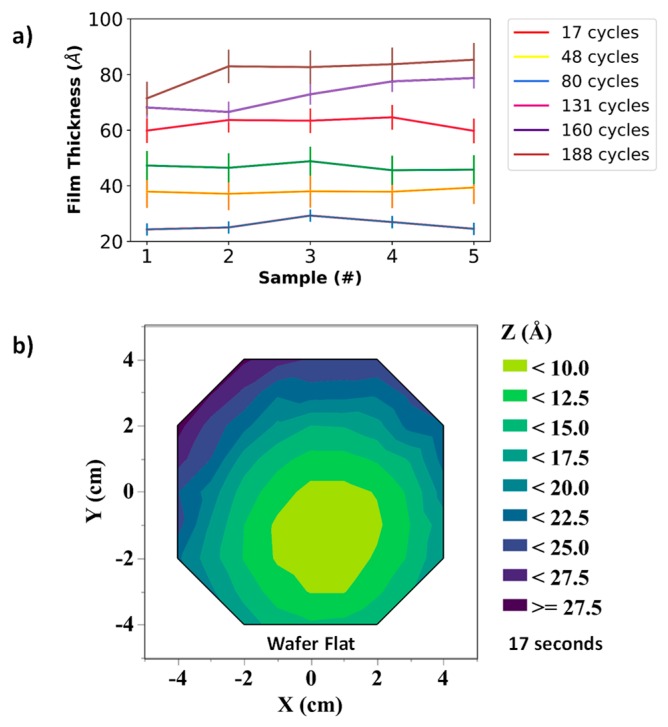
Uniformity measurements of (**a**) SiO_2_ on (**b**) α-Si on a 4-inch wafer.

Figure 5a shows the contour mapping of SiO_2_ film thickness on a 4-inch wafer for six different deposition cycles, and the standard deviation measurements of each of its five repetitive depositions are given in the table. The interpolated contour plots and standard deviation data were obtained from using the JMP statistical analysis software developed by the SAS institute. As shown in Figure 5b, the maximum standard deviation, which is a direct measurement of the film’s non-uniformity, was found to be ~6 Å. Figure 6 shows the variation in film thickness for each repetition. The maximum standard deviation in thickness across multiple depositions was 5.57 Å (for 48 cycles of ALD). Similar experiments were performed to characterize the α-Si film, which was also deposited on a 4-inch oxidized Si wafer. Measurements indicate a standard deviation of 5.73 Å on the sample. These measurements are a clear indication that there is minimal variation in spacer layer thickness. Therefore this fabrication technique can be effectively used for batch fabrication of the nanogap electrodes with sub 10 nm spacer thickness.

### 3.2. SEM Imaging 

**Figure 7:**
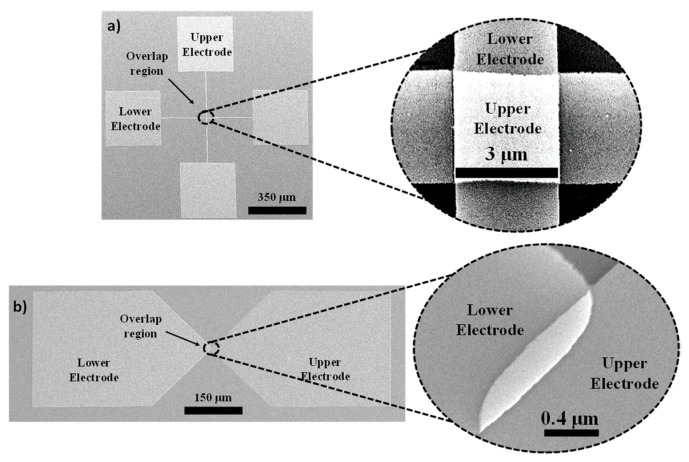
High-resolution SEM images of (**a**) square-overlap layout (**b**) point-overlap layout with zoomed-in images of their overlap regions.

**Figure 8:**
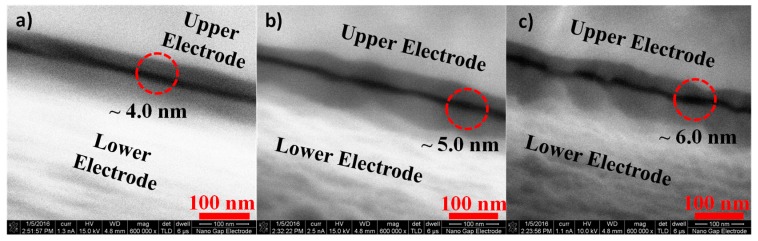
High-resolution SEM images of (**a**) 4.0 nm (**b**) 5.0 nm and (**c**) 6.0 nm gaps.

SEM images of the fabricated square-overlap layout and point-overlap layout devices are shown in Figure 7a,b respectively. The device footprint for the square-overlap and the point-overlap schemes are ~0.36 mm^2^ and 0.27 mm^2^ and a zoomed-in section of their overlap region reveals an area of ~16 µm^2^ and 0.24 µm^2^ respectively. SEM images of the nanogap between electrodes for various spacer thicknesses are shown in Figure 8b–d. As evident in the SEM images, the nanogap thicknesses are in good agreement with the optical measurements discussed in 3.1 and are fairly uniform in nature. 

### 3.3. I–V Characteristics of Square-Overlap and Point-Overlap Layout Devices Across the Wafer

**Figure 9:**
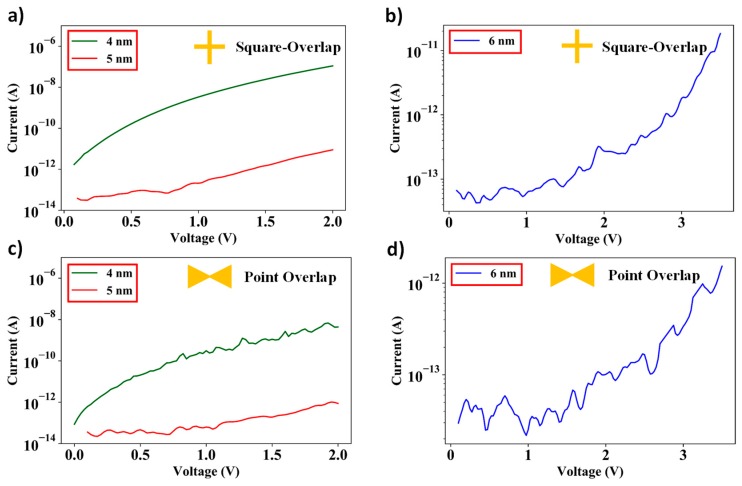
I–V measurements for square-overlap layout having spacer thickness of (**a**) 4 nm and 5 nm, (**b**) 6 nm, and point-overlap layout having spacer thickness of (**c**) 4 nm and 5 nm, and (**d**) 6 nm.

Figure 9a–d shows I–V characteristics of the square-overlap and point-overlap devices respectively for various spacer thicknesses. As is evident from the plots, the current exponentially reduces as the thickness of the spacer layer increases from 4–6 nm. In addition, the junction current is significantly lower for the point-overlap in comparison to the square-overlap layout due to a significant reduction in overlap area. The average junction resistance of the square-overlap layout devices ranged from 3.22 GΩ (for 4 nm spacer layer thickness), 3.83 × 103 GΩ (for 5 nm spacer layer thickness) and 33.3 × 103 GΩ (for 6 nm spacer layer thickness), and the average resistance of the point-overlap layout devices ranged from 25 GΩ (for 4 nm spacer layer thickness), 1 × 104 GΩ (for 5 nm spacer layer thickness) and 46 × 103 GΩ (for 6 nm spacer layer thickness).

**Figure 10:**
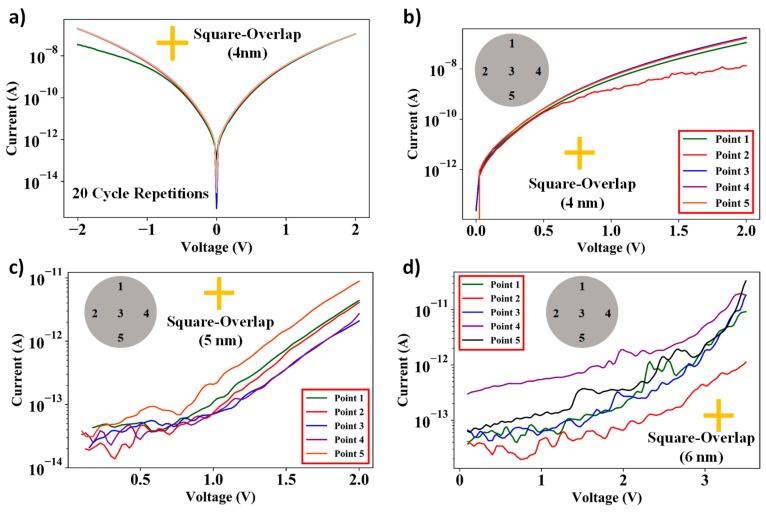
Plots for (**a**) 20 cycles of repetitive I–V measurements of square-overlap layout devices having spacer thickness of 4nm, and I–V measurements of square-overlap devices over five different areas on a 4-inch Si wafer having spacer layer thicknesses of (**b**) 4 nm, (**c**) 5 nm, and (**d**) 6 nm.

Figure 10a shows the I–V characteristics on a square-overlap layout design having spacer thickness of 4 nm for 20 repetitive cycles. As is evident from the plot, there is negligible change in the I–V characteristics even after 20 repetitions of the I–V measurements. Figure 10b–d shows I–V characteristics of the nanogap electrode devices across the wafer. The plots indicate a maximum variation of one order of magnitude in the I–V curves. The reason for that is an exponential dependence of tunneling current on spacer gap. As shown in Figure 5 and Figure 6, the standard deviation of the deposited spacer films is ~0.5 nm. Therefore, the tunneling current is susceptible to a maximum variation of two orders of magnitude across the wafer.

The plot shows that there is no significant change in I–V characteristics over different regions of the wafer. The differences in I–V curves are a result of minor non-uniformities in the thickness and surface defects of ultra-thin films deposited over 4-inch wafers. However, as the plots indicate, this method can be effectively used to batch-fabricate nanogap electrodes having a gap of <10 nm. 

### 3.4. Tunneling Current Measurements and Transition from Direct Tunneling to Fowler–Nordheim Tunneling

**Figure 11:**
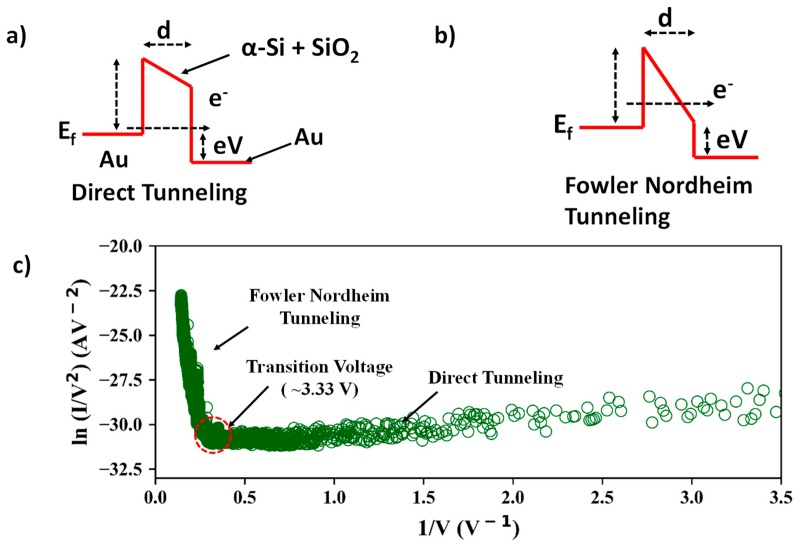
Potential barrier shapes for (**a**) direct tunneling and (**b**) the Fowler–Nordheim tunneling regime, (**c**) transition from the direct tunneling regime to the Fowler–Nordheim tunneling regime upon increase in biasing voltage.

Different charge transport phenomena can occur across a junction barrier as a function of barrier height and thickness, operating temperature, and biasing voltage. At low junction widths and very low biasing voltages, if the temperature is sufficiently high, classical charge transport can occur. Charge carriers can overcome the barrier potential because of thermal energy at high ambient temperatures. This regime of conduction is the “thermionic emission” [25]. When the ambient temperature is low, there cannot be any classical charge transport across the barrier. However, with an increasing biasing voltage, the barrier potential becomes approximately trapezoidal and “direct quantum tunneling” is the dominant charge transport phenomenon across the barrier potential [25]. When the bias voltage is increased further, the barrier potential becomes approximately triangular and the charge transport phenomenon is described by Fowler-Nordheim (F–N) tunneling [25]. The differences in barrier shape between the direct tunneling and Fowler–Nordheim tunneling regimes are shown in Figure 11a,b. 

Tunneling I–V characteristics of an Metal-Insulator-Metal (M-I-M) device can be expressed by the following equations [26,27]: $I=V\times exp^{-\left(\frac{2d\sqrt{2m\Phi }}{\hbar }\right)}$(for *V* < *V_trans_*: direct-tunneling) and $I=V^{2}\times exp^{-\left(\frac{4d\sqrt{2m\Phi^{3}}}{3\hbar eV}\right)}$ (for *V* > *V_trans_*: Fowler–Nordheim tunneling), where *d* is the insulator layer thickness, *e* is the charge of an electron, *m* is the effective mass of an electron, *Φ* is the potential barrier height, *ħ* is the reduced Planck’s constant, and *V_trans_* is the voltage at which the tunneling current regime changes from direct-tunneling to Fowler–Nordheim tunneling, and it is approximately $V_{trans}=2/3\cdot (\Phi /e)$. For the same current level, at larger thicknesses the F–N current becomes dominant because *V > V_trans_*.

Current conduction across the composite stack layer of partially etched away α-Si and SiO_2_ can be modelled using Simmons’ approach for any arbitrary barrier shape [28]. It has been demonstrated by Joshi [29] and Ikuno [26] that the potential barrier height of a dielectric film can be deduced by plotting the transition of the tunneling current regime from direct tunneling to Fowler–Nordheim current. Figure 11c shows the transition from direct tunneling to Fowler–Nordheim tunneling at ~0.28 (V-1) or ~3.5 V, which is the transition voltage for devices having a spacer thickness of 6 nm. Therefore the spacer stack of the device has a potential barrier of ~3.5 eV. This value is similar to the results obtained by Joshi [29] for SiO_2_ films. The linear slope of the graph beyond the threshold voltage and logarithmic slope at voltages lower than the threshold clearly indicate that direct tunneling occurs at low bias voltages and Fowler–Nordheim tunneling occurs at higher biasing voltages through the composite spacer stack of the fabricated device. 

### 3.5. Dielectric Breakdown Measurements and I–V Measurements Across Various Temperatures

**Figure 12:**
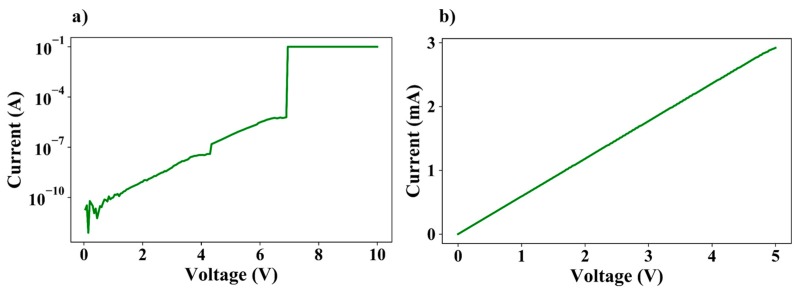
(**a**) Instantaneous breakdown of the dielectric film at ~7 V demonstrated by a sudden increase in conduction current. (**b**) I–V plot of the device after dielectric breakdown typical of ohmic conduction.

Dielectric breakdown refers to the dielectric layer losing its insulating properties and becoming electrically conductive and is one of the major causes of device failure in the semiconductor industry. Therefore, one of the critical parameters of device characterization is the dielectric lifetime. There are mainly two failure modes observed in thin films [30]. The first is instantaneous breakdown (where the charge transport across the dielectric junction instantaneously rises very sharply when the biasing voltage reaches a critical level) and the second is time-dependent dielectric breakdown or TDDB (where the eventual breakdown of the insulating film after a specific duration of time results from a continuous charge transport across the junction). Since we are mainly concerned with determining the maximum operating voltage of the device, in this paper, we limit our discussion to instantaneous failure of the dielectric thin film. Once the breakdown voltage is applied across the device electrodes, we observe an irreversible degradation of the spacer film. Figure 12a shows the I–V characteristics of a fabricated ~6.0 nm spacer layer nanogap device, measured in atmospheric conditions and at room temperature. As evident from the plot, at ~ 7 V the current flowing across the device suddenly jumps to a much higher value, thereby indicating an instantaneous breakdown of the dielectric layer. Figure 12b shows the I–V curve of the same device, after dielectric breakdown has been observed. The plots suggest that the dielectric film is now irreversibly damaged and therefore highly conductive, displaying typical ohmic behaviour. 

**Figure 13:**
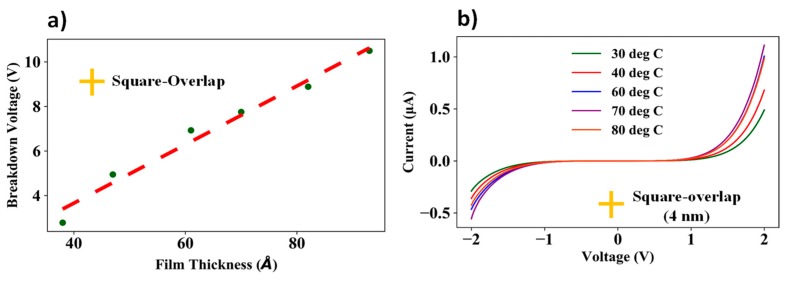
(**a**) Experimentally determined breakdown voltage vs. spacer film thickness (4–9 nm) for square-overlap layout and (**b**) I–V characteristics of 4 nm spacer thickness square-overlap layout for different temperatures.

Figure 13a shows an almost linear dependence of the experimentally determined breakdown field value of the nanogap electrode device on spacer film thickness. The experimental data suggests that the maximum voltage which can be applied across the nanogap electrodes ranges from 2.9 V for the 4.0 nm spacer layer to 10.2 V for the 9.0 nm spacer layer, suggesting that the spacer film consisting of ∝-Si and SiO_2_ has an average breakdown field value of ~11.0 MV/cm. Since the ∝-Si is ultra-thin, it can be assumed to be highly conductive. Therefore, it is the dielectric component of the spacer film (SiO_2_) which degrades irreversibly. According to the thickness calibration curve of the SiO_2_ films given in 4b and the breakdown plot in 13a, the breakdown field of the dielectric layer was determined to be 13–14 MV/cm, which is in course agreement with the experimentally determined results shown by Usui et al. [31]. The minor differences in breakdown field values can be attributed to non-uniformities in the deposited films.

Figure 13b shows the I–V traces for the square-overlap layout device having a 4 nm spacer for different heating temperatures from 30 degrees Celsius to 80 degrees Celsius. As shown in the plot, for bias voltages between –1 to +1 V, since the current conduction is mainly through tunneling, it is fairly independent of operating temperature. However, for bias voltages <1.5 V and >1.5 V, the current conduction typically resembles Schottky current emission, where the I–V characteristics are dependent on the operating temperature. 

Preliminary sensing results have demonstrated that these nanogap electrodes can be effectively used as chemiresistors for label-free electrical detection of both gas and proteins after chemical functionalization. For demonstration purposes, we have selected 1,5-diaminopentane (commonly known as cadaverine) as our target gas and Carbonic anhydrase 2 (CA-II) and Bovine Serum Albumin (BSA) as target proteins [15,17]. The junction resistance, which is typically in the order of G-Ω, drops by several orders of magnitude upon detection of the chemical agent, showing successful resistance switching at the molecular scale.

## 4. Conclusions

We fabricated gold nanogap tunneling electrodes with a spacer thickness as low as 4.0 nm and performed extensive characterization of the spacer layer and the device. Optical measurements revealed an average non-uniformity of 0.46 nm in the SiO_2_ film and ~0.58 nm in the α-Si film. Deposition rates were found to be ~0.7 Å of SiO_2_ per cycle of plasma-enhanced ALD at 200 °C and a deposition rate of ~0.9 Å per second for sputtered α-Si. I–V characteristics showed that the fabricated devices demonstrated a maximum DC resistance of 46 × 10^3^ GΩ between the electrodes, which is an extremely high off-resistance for switching applications. Repetitive I–V measurements on a single device showed negligible drift in electrical characteristics. Electrical measurements performed on devices across the wafer displayed some non-uniformities in electrical properties which is a direct result of very minor fabrication errors and exponential dependence of tunneling current on spacer thickness. These non-uniformities are in accordance with the extensive uniformity measurements performed on the spacer layers. Therefore the fabrication method can be used to batch-fabricate nanogap electrodes having sub-10 nm spacer thickness. Tunneling current measurements demonstrated the presence of both direct tunneling and Fowler–Nordheim tunneling regimes depending on the biasing voltage. Fowler–Nordheim plots revealed that the barrier potential for the spacer layer is ~3.5 eV. Breakdown measurements showed that the average breakdown field for the fabricated devices was 11 MV/cm. I–V measurements at different heating temperatures also displayed electrical conduction which is typical of Schottky emission. Preliminary results have already been shown where nanogap sensors fabricated using this technique have been used as chemiresistors for the near reversible detection of cadaverine gas, BSA and CA-II proteins. Therefore, these devices are ideal candidates for sensor nodes in Internet of Things (IOT) applications and low-power sensing.
